# Addressing multi-region data linkage needs through data sharing agreements – Three Canadian initiatives.

**DOI:** 10.23889/ijpds.v7i3.2093

**Published:** 2022-08-25

**Authors:** Donna Curtis Maillet, Ted McDonald

**Affiliations:** 1 New Brunswick Institute for Research, Data and Training, University of New Brunswick

## Objectives

Data sharing and administrative data access for multi-jurisdictional research must accommodate all local requirements for the protection of personal information and personal health data. Navigating the different safeguards for data sharing found in all relevant Canadian provincial and territorial legislation requires exploring innovative, privacy compliant solutions.

## Approach

Health Data Research Network Canada (HDRN Canada) and one of its provincial data centre partners, New Brunswick Institute for Research, Data and Training (NB-IRDT), are undertaking unconventional approaches to data sharing that balance legislative compliance and data access needs. Three approaches are being pursued: harmonizing data sharing agreements between a national longitudinal study and 10 provincial data centres; facilitating 4 individual data sharing agreements with one regional cohort of a nationwide study; and coordinating data sharing between 4 provincial government departments and one national data centre. Each approach has unique features that presented problems needing innovative resolutions.

## Results

The experiences and challenges of these three approaches to data sharing for multi-regional research have been perplexing but ultimately are leading to productive outcomes. Learnings acquired enable partners to move beyond anecdotal perceptions of data sharing limitations to practical solutions that can be applied in future data sharing partnerships.

The ongoing project management approach of these initiatives identified a core set of somewhat predictable devices to ensure the advancement of simultaneous multiple regional data sharing agreements. These include the assignment of a project coordinator role, clear and ongoing communication, and the ability to negotiate and manage expectations. More innovative, however, is the roadmap that evolved, laying out the necessary (previously unanticipated) measures and sequence in which they should occur for the most expedient outcome.

## Conclusion

To harness the potential of multi-regional research in Canada, provinces and territories must identify feasible and efficient data sharing solutions. The identification of adoptable and replicable legislatively compliant data sharing practices will increase potential data accessibility. This will increase available data and data platforms for research informed policy decisions.

